# Pre-lacteal feeding practice and associated factors among mothers having children less than two years of age in Aksum town, Tigray, Ethiopia, 2017: a cross-sectional study

**DOI:** 10.1186/s12887-018-1284-7

**Published:** 2018-09-25

**Authors:** Girmay Tekaly, Mekuria Kassa, Tilahun Belete, Hagos Tasew, Tekelwoini Mariye, Tsega Teshale

**Affiliations:** 1 Department of Pediatrics and Child Health Nursing, School of Nursing, College of Health Science, Aksum University, Aksum, Ethiopia; 2 Department of Nursing, College of Health Science, Mekelle University, Mekelle, Ethiopia; 3 Department of Adult Health Nursing, School of Nursing, College of Health Science, Aksum University, Aksum, Ethiopia; 4 Department of Medical Laboratory, College of Health Science, Aksum University, Aksum, Ethiopia

**Keywords:** Pre-lacteal feeding, Mothers, Children less than two years, Aksum town

## Abstract

**Background:**

Pre-lacteal feeding has continued as a deep-rooted nutritional malpractice in developing countries. Pre-lacteal feeding is a barrier to the implementation of optimal breastfeeding practices and increases the risk of neonatal early-life diseases and mortality. Therefore, the aim of this study was to assess pre-lacteal feeding practice and associated factors among mothers having children less than 2 years of age in Aksum town, central Tigray, Ethiopia.

**Methods:**

A community-based cross-sectional study was conducted to interview 477 mother-child pairs by systematic random sampling technique. Data were collected through interviewer-administered semi-structured questionnaires. Data were coded, entered, cleaned and edited using EPIDATA version 3.1 and export to SPSS Version 22.0 for analysis. To identify the significant variables binary logistic regression were employed. Variables with *p*-value < 0.05 at 95% CI in multivariate logistic regression were considered statistically significant.

**Result:**

The prevalence of pre-lacteal feeding in Aksum town was 10.1% (95% CI: 7.3%, 13%). Mothers with no previous birth (AOR: 2.93(95% CI:1.21,7.09)), birth spacing less than 24 (AOR: 2.88(95% CI: 1.15,7.25)), colostrum discarding (AOR: 6.72 (95% CI: 2.49,18.12)), less than four anti natal care follow up (AOR: 10.55 (95% CI: 4.78,23.40)), those who underwent cesarean section (AOR: 4.38 (95% CI:1.72,11.12)) and maternal believe on purported advantage of pre-lacteal feeding (AOR: 3.36 (95%CI: 1.62,6.96)) were more likely to practice pre-lacteal feeding to their infants.

**Conclusions:**

Pre-lacteal feeding is still practiced in the study area. Childbirth spacing, colostrum discarding, antenatal Care follow up, maternal belief in pre-lacteal feeding was contributing factors for practicing of pre-lacteal feeding. Coordination and sustaining the existing strategies and approaches are recommended to give emphasis on the nutritional value of colostrum and anti-natal care follow up.

## Background

Globally, it is estimated that every day about 4000 infants and young children die worldwide because they don’t breastfeed [1]. Of around 3 million neonatal deaths every year, two-thirds occur in South-East Asia and sub-Saharan Africa [2]. Sub -Saharan Africa, still with the highest neonatal mortality rates in the world [3].

A pre-lacteal feeding (PLF) is any food except mother’s milk provided to a newborn before initiating breastfeeding in the first 3 days of life [4, 5]. The most common pre-lacteal foods given to infants in many low-middle income countries could be grouped into three: water only, water-based (rice water, herbal mixture, juice), and milk-based (animal milk, infant formula) [6]. Water is dangerous pre-lacteal feed in terms of the detrimental effect on the nutritional aspect and makes the neonate more prone for early risk of severe gastrointestinal infections [7].

Pre-lacteal feeding is a major barrier to first fundamental rights of exclusive breastfeeding (EBF) [8, 9]. The practice of giving other substances (pre-lacteal feeding) to the newborn babies even before lactation is a common cultural practice and this practice also delays the initiation of breastfeeding [10]. pre-lacteal feeding is a risk indicator for infant morbidity and mortality especially during the neonatal period and Some of the practices of pre-lacteal feeding are associated with different belief, misconceptions, faith, and advice by the senior family members or priests of some religions [7].

The child is vulnerable in nutrition, socioeconomic and health factors, which causes malnutrition [11]. Malnutrition is an underlying factor in more than 50% of the major cause of infant mortality and the risk of malnutrition in children during the first 2 years of life is an indication of poor infant feeding practices [12]. Poor feeding practices are chief challenges to the social and economic development of one country [11].

Pre-lacteal feeding practice deprives newborns of colostrum rich in nutrients and immunoglobulins—thus, causing a reduction of the priming of the gastrointestinal tract, and increases the risk of infant morbidity and mortality [13]. Colostrum deprivation was the major cause of stunting in children [14].

Pre-lacteal feeding and its consequences contribute to significant health problems, poor intellectual, physical development and lowered resistance to diseases [15]. In addition, mother-baby bonding may be interrupted by pre-lacteal feeding as it decreases skin-to-skin contact. Thus, this feeding process reduces the practice of exclusive breastfeeding which can be dangerous to the child and may even result in death [16].

Even though, Ethiopia has developed the National Infant and Young Child Feeding (IYCF) Guideline [17] and acknowledged gains of Baby Friendly Hospital Initiative (BFHI) that discourages pre-lacteal feeding practices on newborns to achieve optimal breastfeeding [18], a wide range of harmful newborn feeding practices are documented.

This study will help to health care service provider in their counseling/health education session. This also helps for policymakers, Non-Governmental Organizations (NGOs) and other stakeholders to formulate appropriate implementation tool for achieving sustainable development goal. Moreover, the finding of this study will also help as a baseline data for researchers for further research with this regard. The purpose of the study was to assess pre-lacteal feeding practice and associated factor among mothers having children less than 2 years of age in Aksum town, Central Tigray, Ethiopia.

## Methods

### Study design and setting

A community-based cross-sectional study design was employed in Aksum town of northern Ethiopia from March 1 to 30/ 2017. Aksum town is located 1024 Km north of Addis Ababa and 241 Km far from Mekelle which is the capital city of Tigray region. According to the Central Statistical Agency of Ethiopia (CSA), the population of the town was 56,576 [19, 20]. According to Aksum town health office, the town has one general hospital, one referral hospital, two health center and seven private clinics.

### Sample size determination

The sample size was determined based on the formula used to estimate a single population proportion by using 24.4% prevalence of PLF in Fitche town, north Showa, Ethiopia [21] and a 5% margin of error with 95% confidence level.

$$ \mathrm{n}=\frac{{\left(\mathrm{z}/2\ \mathrm{a}\right)}^2\mathrm{p}\ \left(1\hbox{-} \mathrm{p}\right)}{{\mathrm{d}}^2}=\frac{(1.96)^20.244\left(1\hbox{-} 0.244\right)}{(0.05)^2}=283 $$

The required final sample size with and a design effect of 1.5 and adjustment for non-response rate (15%) was 489.

### Study population

Mothers having children less than 2 years of age who were living for ≥6 months in the selected kebeles of Aksum town were considered as the study population. Mothers who live < 6 months in the town and non-biological mothers were excluded from the study.

### Sampling technique

Multi-stage sampling technique was employed to select 489 study participants. A pre-survey was conducted before the actual day of data collection and 5629 mother-child pairs were targeted in the selected five kebeles (the smallest administrative unit in Ethiopia). From the total of 5 Kebeles of Aksum town, 3 Kebeles were selected by lottery method. To obtain the sample size from every 3 kebeles proportional allocation to sample size was done. Participating households from the selected Kebele’s were identified using a systematic random sampling technique. Finally, every 9th mother from each Kebeles was identified until the required sample size fulfilled and the starting mother was selected using a lottery method by using the house number.

### Data collection tools and procedure

Data was collected using interviewer-administered semi-structured questionnaires by six diploma midwives and three Bachelor of Science degree holder as supervisors. Data were adapted from Ethiopian Demographic and Health Survey [22], Ethiopian National Nutrition Program [16], from the research done in Raya kobo district [23], Harari region [13], Mizan Aman town [24] and Fitche town [21]. The questionnaire was adapted and contextualized to fit the research objective and the local condition.

### Study variables

In this study, the outcome variable was pre-lacteal feeding practice among mothers of children aged less than 24 months. The independent variables were maternal and child Socio-demographic variable (number of children, family size, birth order, maternal age, educational status, occupation, religion), feeding practice (colostrum avoidance, breastfeeding initiation), health care service utilization (ANC utilization, place of delivery and mode of delivery) and maternal level of information on the risk of pre-lacteal feeding.

### Operational definitions

#### Antenatal care utilization

Having at least one visit to a health institution for checkup purpose during the pregnancy of the index child [25].

#### Good level of information about breastfeeding

Those mothers who told two or more components of breastfeeding counseling during their ANC visit (1. Benefits of breastfeeding 2. positioning of the baby 3. Exclusive breastfeeding 4. Management of breast problem 5. expression of breast milk) [26].

#### Poor level of information about breastfeeding

Those mothers who told one or none components of breastfeeding counseling during their ANC visit (1. Benefits of breastfeeding 2. Positioning of the baby 3. Exclusive breastfeeding 4. Management of breast problem 5. expression of breast milk) [26].

#### Postnatal care utilization

Receiving the care provided to the woman and the index child at least once during the 6 weeks’ period following delivery [26].

#### Pre-lacteal feeding

Defined as giving fluid or semisolid food before breastfeeding to an infant during the first 3 days after birth. A mother who gives any food/fluid without the breastmilk regardless of the frequency is considered as pre-lacteal feeding [7].

### Data quality assurance

To ensure data quality, training and orientation were given for 1 day to data collectors and supervisors by the primary investigator. The questionnaire was initially prepared in English and then translated into Tigrigna version (local language) by different experts of both languages to check its consistency. The questionnaire was pre-tested 2 weeks prior to the actual data collection on 5 % of the sample size in shire town and the necessary amendment was done on the questionnaire per pre-test result. The collected data was reviewed and checked for completeness and consistency by the supervisor and principal investigator on a daily bases at the spot during the data collection time. Finally, data collectors were closely followed by the supervisors and principal investigator.

### Data processing and analysis

The Data was coded, entered, cleaned edited using EPIDATA version 3.1, and then exported to SPSS Version 22.0 for analysis. Binary logistic regression analysis was employed to examine the statistical association between the outcome variable and every single independent variable. Variables which showed statistical significance during bivariate analysis at ≤25% (*p*-value ≤0. 25) were entered into multivariate logistic regression to isolate an independent effect of the predictors by using the backward elimination method. The Hosmer-Lemeshow test was used to check the appropriateness of the model for analysis. Results were presented using tables, figures, and texts. Adjusted odds ratios (AOR) with 95% CI, were estimated to assess the strength of associations and statistical significance was declared at a p-value < 0.05.

## Results

### Socio-demographic characteristics

About 477 mothers having children less than 24 months of age were consented to participate in the study with 97.5%% of response rate. Out of the total respondent, 202(42.3%) were aged from 25 to 29 years old, 319(66.9%) had ≥4 family number. About 291(61%) were housewife by occupation and 393(82.4%) of the mothers were had ≤3 children in number. Out of the total children, about 145(30.4%) were aged less than 6 months with 212(44.4%) birth spacing of greater than 24 months (Table 1).

**Table 1:** Socio-demographic characteristics mothers and child, in Aksum town, central zone of Tigray. Ethiopia 2017

Demographic variables	Frequency	Percentage
Age of the mother (*n* = 477)		
≤ 19	18	3.8
20–24	97	20.3
25–29	202	42.3
30–34	85	17.8
35–39	53	11.1
40–44	17	3.6
≥ 45	5	1
Family size (*n* = 477)		
≤ 3	158	33.1
≥ 4	319	66.9
Level of educational (*n* = 477)		
No education	161	33.8
Primary school (1–8)	120	25.2
Secondary school and above	196	41.1
Marital status (*n* = 477)		
Single	17	3.6
Married	407	85.3
Widowed	43	9
Divorced	10	2.1
Religion (*n* = 477)		
Orthodox	396	83
Muslim	81	17
Ethnicity (*n* = 477)		
Tigrian	473	99.2
Amhara	4	0.8
Occupation (*n* = 477)		
Housewife	291	61
Governmental employee	59	12.4
Private employee	91	19.1
Daily labor	35	7.3
Other	1	0.2
Number of children (*n* = 477)		
≤ 3	393	82.4
≥ 4	84	17.6
Age of the child (*n* = 477)		
< 6 months	145	30.4
6–11 months	141	29.6
12–17 months	89	18.7
18–24 months	102	21.4
Sex of the child (*n* = 477)		
Male	254	53.2
Female	223	46.8
Birth order (*n* = 477)		
Birth order one	150	31.4
Birth order 2–3	239	50.1
Birth order ≥4	88	18.4
Birth spacing (*n* = 477)		
No previous child	150	31.4
< 24 months	115	24.1
≥ 24 months	212	44.4

### Feeding practice in the study population

In this study, about 48 (10.1% (95% CI: 7.3%, 13%)) respondents give pre-lacteal feeding within 3 days before giving breastfeeding to their child. The most common type of pre-lacteal feeding given to the child was formula milk 15 (31.3%). About 16 (33.3%) of the respondents were given pre-lacteal feeding to their child due to breastfeeding problem at the time of childbirth. Regarding the influence/advise to provide such kind of pre-lacteal feeding, mothers own decision was more dominant factor 31 (64.6%). About 271(56.8%) mothers were initiate breastfeeding within 1 h (Table 2).

**Table 2:** Feeding practice of mothers, in Aksum town, central zone of Tigray, 2017

Variables	Frequency	Percentage
Pre-lacteal feeding practice for the index child (*n* = 477)		
Yes	48	10.1
No	429	89.9
Type of pre-lacteal (*n* = 48)		
Formula milk	15	31.3
Plain water	13	27.1
Sugar/glucose water	9	18.8
Caw milk	6	12.5
Butter	3	6.3
Other^a^	2	4.2
Reason to give pre-lacteal (*n* = 48)		
Breast problem	16	33.3
Breast feed for infant will cause thirsty	13	27.1
Maternal medical illness	10	20.8
For child growth	3	6.3
Infant feeding problem	3	6.3
Inadequate breast milk secretion	2	4.2
Cultural practice	1	2.1
Influence to give pre-lacteal feeding (*n* = 48)		
Mothers own decision	31	64.6
Health professional	13	27.1
Traditional birth attendant	3	6.3
Grand mothers	1	2.1
Colostrum giving (*n* = 477)		
Yes	447	93.7
No	30	6.3
Reason for discarding colostrum (30)		
Maternal medical illness	12	40
My breast has no milk	12	40
For the child growth	4	13.3
Cause abdominal discomfort and diarrhea	2	6.7
Breast feeding initiation (477)		
Within 1 h	271	56.8
Greater than 1 h	206	43.2

^a^Tenadam with water

### Maternal health care service utilization

From the total 461(96.6%) respondent mothers who were attended ANC visit; 341(71.5%) utilized four times and above (which is internationally recommended) and 467(97.9%) had gotten breastfeeding counseling at ANC clinic. From these who had gotten breastfeeding counseling at ANC clinic, 228(48%) of them were counseled about the benefit of breastfeeding. Four hundred fifty-three (95%) mothers were delivered their child at governmental institutions with 436(91.4%) of them were delivered through normal spontaneous delivery and all facility delivery was assisted by a health professional. About 412(86.4%) mothers had at least one visit of PNC and all of them were got breastfeeding counseling in the post-natal clinic (Table 3).

**Table 3:** Maternal health care service utilization of mothers, in Aksum town, Tigray, Ethiopia 2017

Variables	Frequency	Percentage
ANC visit (*n* = 477)		
Yes	461	96.6
No	16	3.4
How many (*n* = 477)		
> =4	341	71.5
< 4	120	25.2
Not at all	16	3.4
Breast feeding counseling (*n* = 477)		
Yes	467	97.9
No	10	2.1
Place of giving birth (*n* = 477)		
Health facility	453	95
At home	24	5
Mode of delivery (*n* = 477)		
C/S delivery	41	8.6
Normal spontaneous	436	91.4
The person who assisted you during delivery (*n* = 477)		
Health profession	453	95
Traditional birth attendant	24	5
PNC follow (*n* = 477)		
Yes	412	86.4
No	65	13.6

### Maternal level of information on pre-lacteal feeding

Of the total 477 respondents, 447 (93.7%) respondent mothers had information on the advantage of colostrum giving to their child. About 434 (91%) mothers were at the good level of information by which they were able to mention two or more components of breastfeeding counseling during their ANC visit. In this study 165 (34.6%) mothers believe in the purported advantage of pre-lacteal feeding. Of these 101 (61.2%) respondents believe that pre-lacteal feeding was important for child health and growth. About 376 (78.8%) mothers were having information on the risk associated with giving of pre-lacteal feeding to the infant. The problems associated with pre-lacteal feeding includes 343 (72.4%) diarrhea and vomiting and 274 (41.5%) (Table 4).

**Table 4:** Maternal level of information on pre-lacteal feeding among mothers having children less than 24 months, in Aksum town, Tigray, Ethiopia, 2017

Variables	Frequency	Percentage
Advantage of Colostrum (*n* = 477)		
Yes	447	93.7
No	30	6.3
Level of information (*n* = 477)		
Poor level	43	9
Good level	434	91
Believe on purported PLF advantage (*n* = 477)^a^		
Yes	165	34.6
No	312	65.4
Reason for believing on purported advantages (*n* = 165)^b^		
For child health and growth	101	61.2
Breast feed to child cause thirty	53	32.1
To calm the baby	21	12.7
To clean infant’s bowel/throat/mouth	7	4.2
Other^c^	1	0.6
Risk of PLF (*n* = 477)		
Yes	376	78.8
No	101	21.2
Information on risks of PLF (*n* = 376)^b^		
Diarrhea & vomiting	343	72.4
Poor growth	278	41.5
Infection	303	36.6

^a^The medical community defines pre-lacteal feeding as (potentially) dangerous which had no any recognized benefits [39], ^b^multiple answer were possible, ^c^culture

### Factors associated with pre-lacteal feeding practice

In the binary logistic regression at *p*-value of **≤**0.25 maternal education, age of the child, birth order, birth spacing, family size, colostrum discarding, number of ANC visit, breastfeeding counseling during ANC visit, place of delivery, mode of delivery, PNC follow up, maternal belief on the purported advantage of pre-lacteal feeding and information on risk associated with pre-lacteal feeding were statistically associated with pre-lacteal feeding.

In multiple logistic regression by using backward elimination method, mothers with no previousthe birth was about three times higher to introduce pre-lacteal feeding than those mothers who with a birth spacing of greater than or equal to 24 months (AOR: 2.93; 95%CI (1.21, 7.09)). A child who born with a birth spacing of less than 24 months were almost three times more likely to practice pre-lacteal feeding than those who born with a birth spacing of greater than or equal to 24 months (AOR:2.89; 95% CI (1.15,7.25)). A child whose mother discarded her colostrum was about seven times higher to receive pre-lacteal feeding (AOR: 6.72; 95% CI (2.49, 18.12)) than those who gave colostrum to their child. Mothers who have an ANC follow up of less than four times were about 11 times higher to give pre-lacteal feeding than mothers who have four and above ANC follow up (AOR: 10.55; 95%CI (4.76, 23.40)). Mothers who underwent cesarean section were about four times higher to practice pre-lacteal feeding as compared to those who delivered through spontaneous vaginal delivery (AOR:4.38 95% CI (1.72,11.12)). Mothers who believe on the purported advantage of pre-lacteal feeding were three times more to give pre-lacteal feeding than those who didn’t believe the advantage of pre-lacteal feeding (AOR:3.36;95%CI (1.62,6.96)) (Table 5).

**Table 5:** Factors associated with pre-lacteal feeding practices among mothers, in Aksum town, Tigray, Ethiopia 2017

Variables	Pre-lacteal feeding		Crude OR (CI: 95%)	Adjusted OR (CI: 95%)
	Yes	No		
Level of education				
No education	29(18%)	132(82%)	3.695(1.78,7.66)	2.4 (0.95,5.93)
Primary school	8 (6.7%)	112(93.3%)	1.2(0.47,3.08)	1.145(0.39,3.38)
Secondary and above	11(5.6%)	185(94.4%)	1	1
Child age				
< 6 months	16(11.3%)	126(88.7%)	1	1
6–11 months	12(8.7%)	126(91.3%)	0.75(0.34,1.65)	0.43(0.16,1.19)
12-17 months	5(5.8%)	81(94.2%)	0.48(0.17,1.36)	0.434(0.13,1.41)
18–24 months	15(14.9%)	86(85.1%)	1.39(0.65,2.96)	0.843(0.31,2.25)
Birth spacing				
No previous child	19(12.7%)	131(87.3%)	2.417(1.14,5.15)	2.931(1.21,7.09) *
< 24 months	17(14.8%)		2.891(1.33, 6.29)	2.887(1.15,7.25) *
≥ 24 months	12(5.7%)	200(94.3%)	1	1
Colostrum giving				
Yes	32(7%)	425(93%)	1	1
No	16(80%)	4(20%)	10.629(4.78, 23.61)	6.724(2.49,18.12) *
Number of ANC visit				
≥ 4	10(2.9%)	331(97.1%)	1	1
< 4	38(27.9%)	98(72.1%)	11.529 (5.44, 24.41)	10.549 (4.76, 23.40) *
Breast feeding counseling^a^				
Yes	45(9.6%)	422(90.4%)	1	1
No	3(30%)	7(70%)	4.019(1.00, 16.09)	0.648(0.07,6.2)
Place of delivery^a^				
Heath facility	40(8.7%)	418(91.3%)	1	1
At home	8(42.1%)	11(57.9%)	5.162(2.08, 12.81)	1.192(0.03,4.77)
Mode of delivery				
Normal	37(8.5%)	399(91.5%)	1	1
C/S	11(26.8%)	30(73.2%)	3.954(1.83,8.53)	4.377 (1.72,11.12) *
PNC follow up^a^				
Yes	36(8.7%)	376(91.3%)	1	1
No	12(18.5%)	53(81.5%)	2.365(1.16, 4.83)	1.323(0.47,3.70)
Believe on purported advantage of PLF				
Yes	30(18.2%)	135(81.8%)	3.63(1.95, 6.81)	3.359 (1.62,6.96) *
No	18(5.8%)	294(94.2%)	1	1
Risk of PLF^a^				
Yes	28(7.4%)	348(92.6%)	1	1
No	20(19.8%)	81(80.2%)	3.069(1.65, 5.72)	1.454(0.58,3.64)
Family size^a^				
< =3	21(13.3%)	137(86.7%)	1	1
> =4	27(8.5%)	292(91.5%)	0.603(0.33,1.10)	1.403(0.35,5.67)

*Statistically significant variables at *p*-value of < 0.05

^a^Variable excluded after adjusting them in multivariate logistic regression

## Discussion

With the existing strategies and approaches which increase the awareness of mothers, there is has poor maternal knowledge of the advantage of pre-lacteal feeding. Generally, there is a relationship between ANC follow up, colostrum discarding, childbirth spacing and mode of delivery with the introduction of pre-lacteal feeding.

This study revealed that the prevalence of pre-lacteal feeding was 10.1%. This is lower than the national prevalence which was 27% [22]. This result was also lower than the study done in selected regions of Ethiopia, which was 28.9% [27]. This could be due to the study participant were from the town and nearby to health institution, they would have more information on the advantage of visiting antenatal/maternal and child health (MCH) clinics and may have better access to health education materials supportive to decrease the pre-lacteal feeding practice [6]. This could be due to the expansion of community health education in the town through the effective information, education and communication (IEC) strategies. The other possible reasons could be due to the difference in year of the study and sample size difference.

This study is also lower than studies done in different corners of Ethiopia. Eastern Ethiopia, which was 45.4% [13], Raya Kobo district 38.8% [23], northwest Ethiopia 26.8% [26], southern Ethiopia 41% [28], north Showa 24.4% [21], South-west Ethiopia 21.9% [24] and Jimma zone 17% [15]. The difference between these studies might be due to the difference in traditional practice between ethnic groups. This difference might also be due to the difference in the study setting, in the case of the Raya Kobo district 86% of the study subjects were from rural areas, whereas in this study the study participants were from the urban part of Aksum town. This might have been the result of key messages on infant feeding being delivered to pregnant women by healthcare workers during the mothers’ attendance at antenatal care. Hence, mothers who reside in the towns have better access to maternal and child health services. Mothers who live in urban has a good coverage of television and newspapers for access to health education and information. The result of this study was comparable with the study done in northeastern Ethiopia 11.1% [29], in Nigeria 11.7% [30] and in India in Gautam Nagori 10.2% [31].

The current finding is also lower than reports from other developing countries (26.5% in Nepal [6], 31.3%, in Uganda [32], 58% Egypt [33] and 88% in India [34]). This could be due to the difference in contextual regions and health policy, our country currently implementing which is mainly focused on prevention with community involvement on different health issues (with special attention to mothers and infants) through implementing a health extension program that works with the health development army and women networking comprised of the community.

Colostrum feeding provides newborns with immunity to infection. Mothers who discard colostrum in the first 5 days were about seven times more likely to practice pre-lacteal feeding than those who give colostrum to their index child. This result is consistent with the study done in northeastern Ethiopia [29]. This might be because those mothers may believe that pre-lacteal feeding has some advantages and/or have cultural practice to feed other than breast milk, thus more likely to feed pre-lacteals. Lack of full information on the advantages of giving newborn colostrum and the disadvantage of pre-lacteal feeding could lead to mothers discarding the first milk [35]. A cesarean section may also hamper immediate colostrum feeding due to post anesthesia or postoperative effects [36]. There are also many women say that they have breastfeeding problems. During this interval, babies are likely to feed pre-lacteal feeding.

Antenatal care visit is a best opportunity to promote skilled attendance at birth and to counsel and educate mothers on essential healthy behaviors like newborn feeding. The result of this study revealed that mothers with less than four ANC visit were about 11 times more likely to introduce pre-lacteal feeding than those who had greater than four ANC follow up. This may be due to the fact that those mothers who follow ANC get information on feeding practice of the newborn and infant from the health workers. This is similar to the studies done in the Harari region [13] and south Ethiopia [28]. This is also consistent with the study done in sub-Saharan Africa [37] and in Nepal [6]. This result was inconsistent with the study done in the selected regions Ethiopian [27]. This could be due to the different sample size difference in which our sample size was smaller than the study done in selected regions Ethiopian. Therefore, Coordination, strengthening and sustaining of the existing strategies and approaches to give more emphasize on the nutritional value of colostrum and ANC services utilization is recommended to reduce health problems associated with the introduction of pre-lacteal feeding**.**

Furthermore, first time mothers were more likely to introduce pre-lacteal feeds in this study. The first-time mothers could have less skill and knowledge of newborn care and proper infant feeding practice. They may also rely more on the older women in the household and community who follow the traditional practice [38]. In this study mothers with no previous birth were about three times higher to practice introduction of pre-lacteal feeding. Moreover, pre-lacteal feeding was almost three times higher among mothers who gave birth within 24 years. Short inter-pregnancy intervals are associated with a higher risk of low birth weight, preterm birth and a higher risk of cesarean section. During that time, the neonate may be admitted to an intensive care unit which may hamper the exclusive breastfeeding and leads to practice pre-lacteal feeding.

In this study, pre-lacteal feeding was about four times higher in mothers who delivered through the cesarean section as compared to those who had vaginal delivered. This is consistent with the studies done in Egypt [33], in Uganda [32] and in India [36]. Use of general or spinal anesthesia for cesarean delivery and the trauma during surgery may delay the recovery of mothers. The caretakers then tend to provide alternative feeding to the baby during this period, often on the suggestion of the hospital staff.

The medical community defines pre-lacteal feeding as (potentially)dangerous which had no any recognized benefits [39]. In this study mothers who believe in the purported advantage of pre-lacteal feeding was about three times higher to provide pre-lacteal feeding to their index child. This implies they have poor knowledge of the risk associated with pre-lacteal feeding [23]. This finding is similar to the study done in northwest Ethiopia [26]. Boosting a mother’s knowledge of IYCF is a cornerstone for implementing sustainable strategies to improve appropriate feeding practices [35].

Findings from this study have a substantial contribution to the promotion of optimal breastfeeding practices and the achievement of the sustainable development goal in reducing child mortality in Ethiopia. However, the limitation of this study was that the information obtained from mothers might be subjected to recall bias. Lack of support with qualitative data is also another limitation. Therefore, further follow up research with qualitative support is recommended to understand the relationship between (cesarean delivery, colostrum discarding) and pre-lacteal feeding. The study also shares the limitation of the cross-sectional study design.

## Conclusions

Although Ethiopia has set breastfeeding policies consistent with international recommendations, there are still neonates who receiving pre-lacteal feeding in Aksum town, which leads to decrease exclusive breastfeeding practices in the town. The current study showed that the prevalence of pre-lacteal feeding is still high that remained a challenge for optimal breastfeeding in the town. Childbirth spacing and maternal-related factors were contributing factors for practicing of pre-lacteal feeding.

## Abbreviations

ANC: Antenatal care
AOR: Adjusted odds ratio
COR: Crude odds ratio
IEC: Information Education and Communication
PLF: Pre-lacteal feeding
